# Effects of improved sanitation on diarrheal reduction for children under five in Idiofa, DR Congo: a cluster randomized trial

**DOI:** 10.1186/s40249-017-0351-x

**Published:** 2017-09-19

**Authors:** Seungman Cha, JaeEun Lee, DongSik Seo, Byoung Mann Park, Paul Mansiangi, Kabore Bernard, Guy Jerome Nkay Mulakub-Yazho, Honore Minka Famasulu

**Affiliations:** 1 Korea International Cooperation Agency, 825 Daewangpangyo-ro, Sujeong-gu, Seongnam-si, Gyeongi-do Republic of Korea; 2 Department of Disease Control, Faculty of Infectious and Tropical Disease, London School of Hygiene & Tropical Medicine, Keppel Street London WC1E 7HT, London, UK; 3 Korea Environment Corporation, 42 Hwangyeong-ro, Seo-gu, Incheon, Republic of Korea; 4 Kinshasa University, 18 Denis street, Yolo-Sud Quarter, Kalamu Zone, Kinshasa, Democratic Republic of the Congo; 5 Water and Sanitation for Africa, Ouaga 03Secteur 27, 441, Rue NabaKiibaBoulsa, Ouagadougou, Burkina Faso; 6 Service National d’Hydraulique Rurale, Av. Colonel Lukusa No. 111–112 C/Gombe, Kinshasa, Democratic Republic of the Congo

**Keywords:** Sanitation, Diarrhea, Well-equipped latrine, Sanitation calendar

## Abstract

**Background:**

The lack of safe water and sanitation contributes to the rampancy of diarrhea in many developing countries.

**Methods:**

This study describes the design of a cluster-randomized trial in Idiofa, the Democratic Republic of the Congo, seeking evidence of the impact of improved sanitation on diarrhea for children under four. Of the 276 quartiers, 18 quartiers were randomly allocated to the intervention or control arm. Seven hundred and-twenty households were sampled and the youngest under-four child in each household was registered for this study. The primary endpoint of the study is diarrheal incidence, prevalence and duration in children under five.

**Discussion:**

Material subsidies will be provided only to the households who complete pit digging plus superstructure and roof construction, regardless of their income level. This study employs a Sanitation Calendar so that the mother of each household can record the diarrheal episodes of her under-four child on a daily basis. The diary enables examination of the effect of the sanitation intervention on diarrhea duration and also resolves the limitation of the small number of clusters in the trial.

In addition, the project will be monitored through the ‘Sanitation Map’, on which all households in the study area, including both the control and intervention arms, are registered. To avoid information bias or courtesy bias, photos will be taken of the latrine during the household visit, and a supervisor will determine well-equipped latrine uptake based on the photos. This reduces the possibility of recall bias and under- or over-estimation of diarrhea, which was the main limitation of previous studies.

**Trial registration:**

The study was approved by the Institutional Review Board of the School of Public Health, Kinshasa University (ESP/CE/040/15; April 13, 2015) and registered as an International Standard Randomized Controlled Trial (ISRCTN: 10,419,317) on March 13, 2015.

**Electronic supplementary material:**

The online version of this article (doi:10.1186/s40249-017-0351-x) contains supplementary material, which is available to authorized users.

## Multilingual abstracts

Please see Additional file 1 for translations of the abstract into the five official working languages of the United Nations.

## Background

As of 2010, 1.731 billion episodes of diarrhea in children aged under five were reported globally [1]. Diarrhea killed 0.578 million children, accounting for 9.2% of all child deaths in 2013 [2]. In developing countries, the high prevalence of diarrhea can be attributed to the lack of safe water and sanitation [3]. The world has made huge strides in achieving the MDG target for safe water coverage over the past decade, meeting the target ahead of schedule in 2010. However, sanitation remains an important challenge for the global development agenda, with the coverage still below the target of 77% [4, 5]. An estimated 2.4 billion people still do not have access to improved sanitation facilities in 2015, and of these, around 1 billion people still practice open defecation [6]. In addition, a huge disparity in sanitation coverage exists among regions and between urban and rural areas. In particular, people without access to improved sanitation facilities are concentrated in Southern Asia and sub-Saharan Africa [1]. The situation seems especially dire in sub-Saharan African countries; Southern Asia has increased coverage from 22% in 1990 to 49% in 2015, while sub-Saharan Africa’s coverage has risen from 24% to only 31% over the same period [4–6]. An urban–rural divide in access to sanitation also exists. Today, only 51% of the rural population has access to improved sanitation facilities worldwide, compared with 82% of the urban population [4–6].

In the Democratic Republic of the Congo (DRC), the mortality of children remains stubbornly high. As of 2013, out of 1000 births, 119 children aged under 5 years lost their lives. In particular, diarrheal diseases are the common causes of mortality, being responsible for approximately 11% of child deaths. Of all sub-Saharan African countries, only seven countries have a higher percentage of diarrhea-specific child death than that of the DRC [7]. The country also has lower water and sanitation coverage than other countries in sub-Saharan Africa. In 2012, the DRC was reported to be one of only three countries where less than half of the population had access to safe water sources [5]. Only 16% of total households in the country have access to improved sanitation, with 4% having access in rural areas in comparison with 36% in urban areas. This is even worse than the sub-Saharan African average of 44 and 24% in urban and rural areas, respectively [8].

The majority of previous studies exploring the effect of improved sanitation, however, were observational [9–12]. Furthermore, only a few studies have investigated the net effect of improved sanitation on diarrheal reduction for children aged under five. Cochran Review results [13] showed that previous studies have neither executed a cluster randomized control trial (cRCT) on sanitation intervention nor assessed the effects on intermediate outcomes, such as the presence of flies. The first study that employed a randomized trial to measure the health impact of large-scale sanitation programs [14] could not find any evidence on the protective effects of improved latrines against child diarrhea prevalence. In a similar vein, a recent cRCT study that employed process evaluation [15] could not find any evidence that increased sanitation coverage is effective in preventing diarrhea. However, it is important to note, as the authors did, that in a situation where the coverage of improved sanitation is not adequate, improved sanitation’s protective effects against diarrhea may not be properly observed. The sufficient level of latrine coverage is a necessary condition to determine the effect of improved latrines on diarrhea prevention, given diarrhea’s transmission characteristics and the phenomenon of herd protection [16]. Other recent trials [17–21] also revealed similar limitations with regard to low coverage of improved latrine uptake and use. However, according to Fuller and his colleagues [22–24], a sanitation intervention has the potential to provide herd protection against diarrheal diseases, effectively reducing the prevalence. Their mathematical modeling demonstrated that when their neighbors use improved sanitation, susceptible persons in a household would face a reduced probability of contracting a diarrheal infection regardless of their own sanitation practices [22]. Therefore, more trials should be conducted in contexts where the coverage level is sufficiently high to better measure and quantify the effects of improved latrines.

We aim to investigate the extent to which well-equipped latrines reduce child diarrhea, especially in the conditions where the latrine coverage reached an almost universal level, or the level required to generate herd protection. Improved latrines can prevent transmission of pathogens via flies, contaminated fields and water [25]. If latrines are not properly equipped, used and managed, they can be sources of potential disease transmission [26]. Therefore, reflecting the 5-f diagram, we will focus on which components latrines should have (pit-hole depth, pit-hole cover, cement slab, and hand-washing facility) and how they should be managed (feces around the pit-hole and flies) in order to disrupt disease transmission. As a secondary research question, we will explore the association of children’s diarrheal prevalence with latrine components (pit-hole depth, pit-hole cover, cement slab, and hand-washing facility) and latrine management (feces around the pit-hole and flies).

## Methods/design

### Study setting

**Fig. 1:**
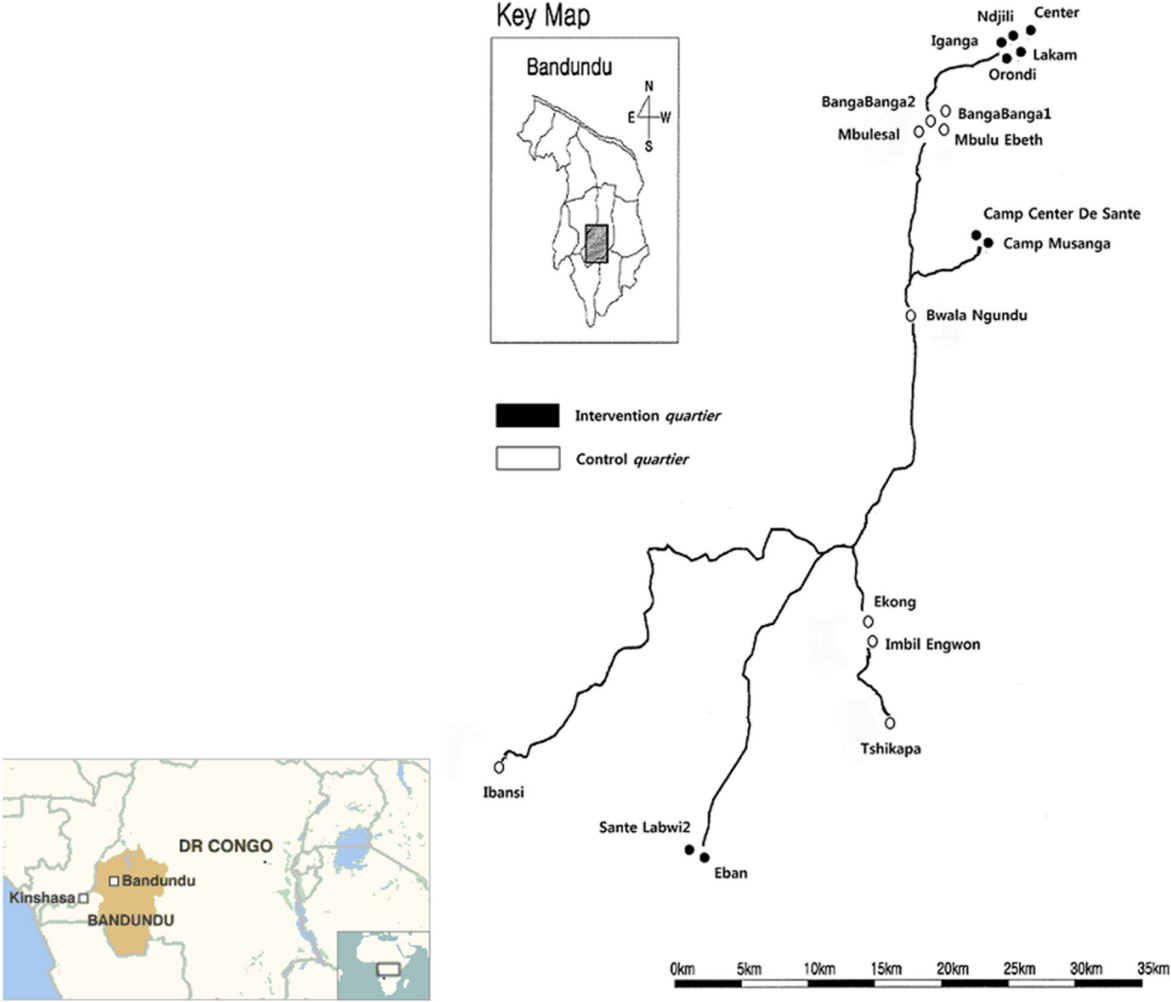
**a** The study area, **b** The study area

The study site is located in the Idiofa Territory of the Kwilu District, Bandundu Province, 655 km away from Kinshasa, the capital city of the DRC (Fig. 1). The territory has five cities and 12 sectors with a population of 1.4 million. Despite the existence of diverse ethnic groups, the Mubunda tribe mainly dominates the territory, with many speaking the Kikongo language. Agriculture is the main source of income in the region.

This water, sanitation and hygiene (WASH) project has been designed to reduce diarrhea among children under four by providing clean water, improving household latrines and promoting relevant hygiene practices. Since the construction and use of latrines by household members cannot be fully achieved without necessary behavioral changes, hygiene promotion was included as one of the activities under the project.

### Study design

This cluster-randomized trial takes a *quartier*, a subdivision of a village, as the randomization unit since well-equipped latrines have an impact on diarrhea transmission across households at a cluster level. A “well-equipped latrine” is defined as having (1) a pit with a depth of more than 1.5 m; (2) a superstructure; (3) a roof; (4) a cement slab; (5) a pit-hole cover; and (6) a hand-washing facility based on the UNICEF Healthy Village Programme in the DRC. Any latrine that does not meet a single requirement will not be defined as a well-equipped latrine in the trial. A *quartier* is the lowest administrative unit in the DRC, where the number of households ranges from 396 to 2873. All the interventions will be undertaken on a *quartier*-wide basis. Since the purpose of the intervention is to reduce diarrhea, a *quartier* will be an appropriate dimension for the transmission zone, where humans, vectors, and intermediate hosts are interacting with each other and sharing a common pool of parasites.

We will employ a phase-in design. For well-equipped latrines, the project will roll out only in an intervention arm for the first phase (8 months). A total of four rounds of household surveys (including the baseline survey) will be conducted every 6 months (June 2015, January and August 2016, and February 2017) throughout the trial. The second round of the survey will be conducted immediately before the control arm will receive the intervention for the next 6 months. After the intervention period, we will have a follow-up period of about 1 year in which the third and the fourth survey will be conducted.

A preliminary survey was conducted from March through May of 2013 to gather information and analyze conditions at the field level to develop the WASH project in the DRC. On the basis of the preliminary results, each *quartie*r was rated by the degree/intensity/severity of the needs in terms of quantity of safe water per person per day *(liter/person/day),* distance to water points from the *quartier* (*km*), and the percentage of households with a household latrine (%).

A two stage cluster sampling method was employed for this study. The 38 target *quartiers* in 12 villages for the project were selected based on the priority scored by degree of need, from which 18 *quartiers* in 10 villages were randomly chosen employing probability proportional to size (PPS). Eighteen *quartiers* were randomly allocated to the intervention or control arm according to the results of the baseline survey, which was conducted in January 2015.

The *quartiers* were allocated into blocks by administrative unit or village according to ‘restricted randomization’.Our study used restricted randomization since it involved selecting randomly from a smaller set of allocations fulfilling certain restrictions. We stratified the *quartiers* into three groups depending on the prevalence of child diarrhea, and within each stratum we grouped the *quartiers* into two for the following reasons: one is to ensure that *quartiers* in the same village be allocated to same arm; the other is to have the same number of *quartiers* for the intervention and control arm.

**Fig. 2:**
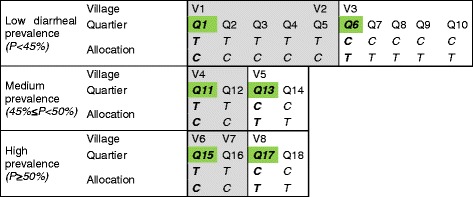
Restricted randomization of the trial. This figure shows how we carried out restricted randomization for the study. We stratified *quartiers* depending on the child diarrheal prevalence, and within each stratum we grouped the *quartiers* into two (shown graded and ungraded in the figure) in order to ensure that *quartiers* in the same villages are allocated to the same arm and also to allocate the same number of *quartiers* to the treatment and control arms. *Quartiers* in the green boxes were selected as representative for each group in each stratum, and the leaders from the *quartiers* participated in the randomization activity, selecting an envelope containing a paper marked O or X without knowing which it would be before they opened it. For instance, if the leader of *Quartier* 1 selected an envelope with O, all the *quartiers* from Q1 through Q5 were to be allocated to the treatment group and Q6 through Q10 were remaining for the control group. Using this method, there were 8 different possible allocation scenarios for the trial. (Village and quartier name) V1: Bangabanga, V2: Bwalenge, V3: KalangandaMukeni, V4: Mayanda, V5: Ingundu, V6: ImpiniNnsi, V7: Punkulu, V8: IntswemLabwi, Q1: Bangabanga1, Q2: Bangabanga2, Q3: MbuluEbeth, Q4: Mbulesal, Q5: Ibansi, Q6: Center, Q7: Lakam, Q8: Ndjili, Q9: Nganda, Q10: Orondi, Q 11: Camp Center de Sante, Q12: Camp Musanga, Q13: Ekong, Q14: ImbilEngwow, Q15: Tshikapa, Q16: BwalaNgundu, Q17: Eban, Q18: Sante Labwi2

A *quartier* was selected as representative for each group in each stratum, and the leaders from the *quartier* participated in the randomization activity to select an envelope containing a paper marked O or X without knowing it. For instance, in Fig. 2, if the leader of *quartier* 1 selected an envelope with O, all the *quartiers* from Q1 through Q5 were to be allocated to the treatment group, and the others from Q6 through Q10 to the control group. Using this method, we could expect 8 different allocation scenarios for the trial.

**Fig. 3:**
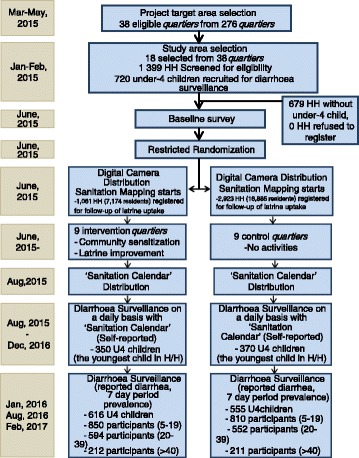
Flow diagram

One of the greatest limitations of restricted randomization lies in the risk that it could bring about biased estimates of effect, especially when there is any difference across the clusters in the possibility of allocation to any given treatment [27]. However, we believe the possibility of a biased result was quite low in our study because there was no difference across *quartiers* in their probability of allocation to the treatment or control arm (Fig. 3).

### Primary health outcome

**Fig. 4:**
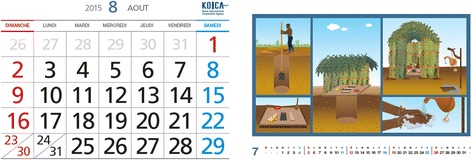
Sanitation Calendar (left: front, right: back)

The primary outcome of the study is reduction in diarrheal incidence (cases per 1000 child-week), prevalence and average duration (days per episode) in children under four. To measure progress, the mother or caregiver of each household will record the diarrheal episodes of the youngest child under four on a daily basis using the ‘Sanitation Calendar’ (Fig. 4). The Sanitation Calendar will enable us to look into the intervention’s effects on incidence density as well as the duration of diarrhea in children under four. A 7-day recall period will be used to measure diarrheal prevalence.

### Eligibility criteria and enrollment

In selecting *quartiers*, we took into account the following eligibility criteria: (1) the lowest coverage of improved sanitation, (2) the lowest coverage of improved water, and (3) no other WASH projects scheduled to roll out from 2015 to 2016.

Households with at least one child under 4 years of age were eligible for this study. The youngest under-four child in each household was registered and assigned an identity number for the longitudinal study, as the follow-up period is about 1 year (Jan. 2016-Feb. 2017) and the cohort would be aging throughout the study. As we will follow an open cohort design, new babies born during the longitudinal survey period are to be enrolled for the study. Child age was verified with a Child Registration Card indicating the birth date of each child. We specifically targeted households with children under five because the majority of morbidity and mortality associated with diarrhea occurs in this group, and thus they are most likely to benefit from the intervention.

A total of 720 households were surveyed out of the 1399 households in the 18 *quartiers* chosen to be the subjects of the study. No household refused to be registered in the study. We obtained informed consent from 720 household heads in a written form. We established three teams, each consisting of six data collectors and one supervisor. A *quartier* was divided into three blocks, and a team was assigned to each block. Each team member started enrolling households from the central location of the block until s/he reached her/his quota or the assigned boundary of the block. In addition, a questionnaire survey was administered for the purpose of identifying WASH status, demographic characteristics and socioeconomic conditions. We achieved a remarkable balance of basic and WASH-related characteristics between the two arms (Table 1).

**Table 1:** Balancing Results

Variable	Intervention	Control	Mean Difference	*P*-value
Diarrhoeal Prevalence (U4Y)	45.9%	45.7%		1.0
Diarrhoeal Prevalence (6–19)	12.4%	11.4%		0.731
Diarrhoeal Prevalence (20–39)	12.4%	14.3%		0.511
Diarrhoeal Prevalence (40~)	4.3%	4.3%		1.000
Latrine coverage (Unimproved)	60.3%	68.0%		0.036
Latrine coverage (Improved)	0%	0%		-
H/H Head gender (male)	88.6%	89.7%		0.719
H/H Head age	40.1(11.64)	40.85(10.92)	0.892	0.401
H/H/H Ethnic group	97.3%	99.4%		0.177
Education level (secondary)	62.4%	62.0%		0.351
Education level (primary)	30.5%	30.9%		0.945
H/H H religion (Christian)	92.4%	97.1%		0.004
Income	25,903 (50,536.12)	23,889 (36,762.34)	2014.44	0.574
H/H members	6.15(2.26)	6.09(2.28)	− 0.060	0.724
No. of U5C	1.66(0.77)	1.59(0.68)	− 0.079	0.146
Mother/Caretaker’s age	30.19(8.96)	31.08(8.96)	0.888	0.192
Youngest under-5 child age (months)	19.83(13.94)	19.95(14.12)	0.122	0.907
Latrine Utilization by all members	6.8%	4.6%		0.413
Main source of water (not-protected)	98.6%	94.6%		0.004
Average time for fetching water (minutes)	115.14(11.07)	94.29(10.51)	− 20.849	0.000
Water quantity (liter)	49.94(52.69)	65.06(51.97)	15.113	0.000
Duration of water storage (days)	2.75(1.56)	2.46(1.25)	− 0.286	0.008
Water container cleaning	96.6%	97.9%		0.478
Water treatment	3.5%	5.1%		0.359
HW Practice (Before eating)	94.5%	96.3%		0.150
HW Practice (After defecation)	86.6%	83.5%		0.719
HW Practice (Before cooking)	61.9%	62.6%		0.469
HW Practice (After cleaning child buttock)	6.7%	8.2%		0.301
HW Practice (After handling a sick person)	17.6%	14.3%		0.426
Latrine type (covering)	5.8%	2.9%		0.170
Latrine type (roof)	86.1%	81.9%		0.254
Latrine type (superstructure)	65.5%	70.6%		0.271
Latrine type (feces)	24.2%	16.8%		0.050
Latrine type (less than 50 cm)	24.7%	19.3%		0.178
Latrine type (flies)	69.5%	59.7%		0.032
Latrine type (flies quantity)	30.3%	19.7%		0.045

### Sample size calculation

Based on the findings of the preliminary survey, we estimated diarrhea prevalence in Idiofa Territory to be 10%. We also made an assumption that the intervention would reduce the prevalence by 2.5% (25% relative reduction) on the basis of systematic reviews. Unlike the previous studies, we calculated sample size on the basis of *Incidence density of diarrhea by ‘child-weeks’* using formula [28] as follows:

The expected value of s^2^ is given by:

E(s^2^) = *λ* Av(1/y_j_) + *σ* ^2^_c_ = *λ*Av(1/y_j_) + k^2^ *λ* ^2^,where λ is the true mean rate, y_j_ is the child-weeks of follow up in the j^th^ cluster, Av(1/y_j_) indicates the mean over all m clusters, σ^2^_c_ is the between-cluster variance of true rates, and k is the coefficient of variation of those rates (22). Using the preliminary survey, we were able to determine that the overall diarrheal rate for the 20 *quartiers* was 0.1 (or 10 per 100 child-weeks). The empirical standard deviation of the observed diarrhea rates was s = 0.051216, and the average of the reciprocal child-weeks per *quartier* was Av(1/y_j_) =0.022222, so that k was estimated as follows:

$$ {\widehat{\sigma}}^2=0.0{51216}^2\hbox{--} \mathrm{0.10.022222}=0.000401,\mathrm{therefore},\mathrm{k}=\surd \left(0.000401/0.1\right)=0.063 $$

With an assumption that the diarrhea rate in control *quartiers* remains constant at λ_0_ = 0.1, we required 80% power (z_β_ = 1.96) if the intervention is to reduce the diarrhea rate by 25% to λ_1_ = 0.10×0.75=.075. Assuming y = 540 child-weeks of observation in each *quartier*, the number of *quartiers* required in each treatment group is given by:

$$ \mathrm{c}=1+{\left(1.96+0.84\right)}^2\left[\left(0.1+0.075\right)/540+{0.063313}^2\left({0.1}^2+{0.075}^2\right)\right]/{\left(0.1-0.075\right)}^2=6.14 $$

Ignoring clustering, y = (1.96 + 0.84)^2^(0.1 + 0.075)/(0.1–0.075)^2^ = 2195.2 child-weeks per group, corresponding to 2195.2/540 = 4.07 *quartiers*. Thus, the expected design effect for this trial would be 6.14/4.07 = 1.51.

Assuming a coefficient of variation of 0.3 with 80% study power and 7.5% diarrheal prevalence in the intervention resulted in seven clusters (7 *quartiers*) per each arm. We increased the number of clusters to nine each in the control and intervention arm, and sampled 360 households in each trial arm. We will follow up at least 12 weeks to make 8640 child-weeks (‘child-weeks’ is the unit of the denominator) in each *quartier* as required to meet 80% power, considering a 12.5% loss to follow-up.

### Intervention

The intervention will employ the approach applied in the Village Assani (Healthy Villages) by UNICEF’s nationwide sanitation program in the DRC in line with the guidelines of the government of the DRC and UNICEF on latrine improvement. The Korea Environment Corporation (KECO) and the Water and Sanitation for Africa (WSA), a Pan-African inter-governmental agency, will implement the project in collaboration with the Service Nationale Hydraulique Rural (SNHR: National Service for Rural Water Supply), DRC. The project is funded by the Korea International Cooperation Agency (KOICA).

### Project implementation

KECO will be responsible for supervision and management of the project implementation. Two sanitation experts have been deployed to this project team from the WSA to provide technical advice and three sanitation officials from SNHR have been seconded to facilitate community mobilization and provide information about the local culture, such as social norms and, if any, taboos during the project period.

Before the intervention, WASH committees were established right after 18 *quartiers* had been selected for the target area of the water supply program in February 2015; therefore, the committees were present in both the intervention and control arms. Nine WASH committee members were elected by the people living in each *quartier*. It was recommended that they elect at least three female members, but several *quartiers* could not recruit at least three female members to form their committee. The main roles and responsibilities of the WASH committee with regard to the water supply program are to maintain water facilities and collect water fees from the *quartier* residents. Later on, after 9 *quartiers* were selected for this sanitation intervention, we added new roles and responsibilities of the committee for latrine improvement for the intervention group and trained the committee members to perform those tasks. The new roles and responsibilities of the WASH committee are defined as (1) community mobilization on latrine improvement; (2) educating community people for healthier hygienic practices; (3) drawing and regularly updating the Sanitation Map; (4) monitoring the progress, especially on the ‘recording status of the Sanitation Calendar’ and ‘latrine improvement situation’ at the household-level in each *quartier*.

**Fig. 5:**
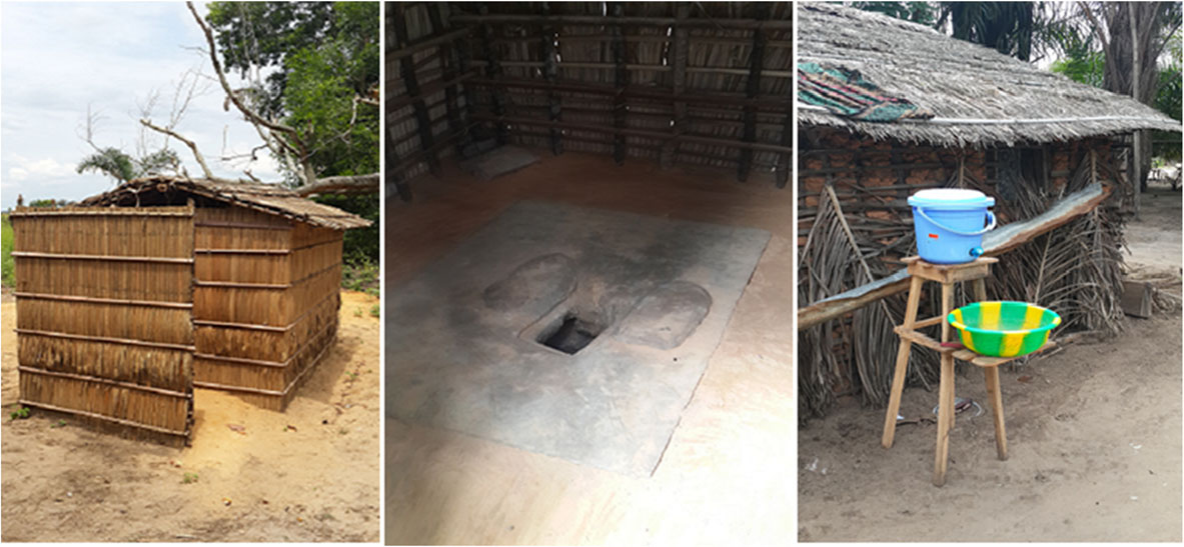
**a** A well-equipped latrine, **b** A well-equipped latrine, **c** A well-equipped latrine

In order to promote increased coverage of well-equipped latrines, material subsidies, cement for making slabs, pit-hole covers and hand-washing facilities (cost US$7.50 per household), will be provided only to the households, regardless of their income level, who complete (1) pit digging and (2) construction of a superstructure and roof. Such conditions for the material support were determined in light of the availability and affordability of a superstructure, roof and pit-hole cover at the local level. The material subsidy is only partial and does not cover the whole cost of construction. The cost of a community member’s labor has been estimated at US$11.62 (10,750 Congolese Franc, exchange rate on July 7, 2015) per each household. According to the survey conducted in the trial, the average monthly income of one person was 21,500 Congolese Francs. The duration of latrine construction for each household is expected to be 10 full days when two people work together (including digging the pit, obtaining timber for the superstructure and thatches for the roof and its installation, molding the slab, making the cover, and installing the hand-washing facility) (Fig. 5).

### Health outcomes assessment, sanitation calendar

The incidence of the diarrhea of the youngest child under four will be recorded by the mother or caregiver on the Sanitation Calendar distributed to each registered household in early August, 2015. The mother or caregiver will be educated to mark ‘O’ whenever her youngest child under four has diarrhea and mark ‘X’ on each day without diarrhea over the next 12-month period of longitudinal observation. Mothers were informed of the definition of diarrhea - three or more of watery stools in 24 h, with pictures of various stool types produced by the World Health Organization.

Households will be monitored by the WASH committee twice a month and instructed to keep recording appropriately. We employed the diary methodology to overcome the limitations reported in previous studies, such as recall bias [29] and reporting fatigue, leading to underestimation [30]. The diary methodology will allow us to capture more precisely the effects of well-equipped latrines on the duration of diarrhea and diarrheal incidence density. As an incentive for keeping a diary, the project has made the Sanitation Calendar a voucher, which can later be exchanged with a gift. During the monthly monitoring visit, the WASH committee will check the status of diary recording by taking a photo of the Sanitation Calendar marked as ‘O’ or ‘X’ for each day of each month.

### Intermediate outcomes: exposure to excreta

#### Sanitary surveys

During the household survey period, data collectors will make an observation on the presence and quantity of feces within a certain distance of the household and *quartier.* The baseline survey conducted in January 2015 revealed that the majority of children under four practiced open defecation. Thus, latrine improvement activities will include the appropriate disposal of human feces, particularly of children and to encourage children to use well-equipped latrines.

### Vectors: fly counts

Previous studies have shown that fly control has a protective effect against diarrhea. We also examine the effect of well-equipped and appropriately managed latrines on fly control. To this end, glue traps will be used to count the number of flies. A sticky trap of the same length will be provided to data collectors so that they can place it around a pit-hole before administering the questionnaire during the household survey. After 30 min, they will check the traps and record the number of flies.

### Data analysis

Intention-to-treat analysis will be conducted to explore how well-equipped latrines reduce diarrhea incidence. To this end, the incidence density of diarrhea in children under four (diarrheal incidence per child-week, per child-month) and the reported diarrheal prevalence of the 7-day period will be calculated. Also, for other age groups (6–19, 20–39, 40 years and above), the reported diarrheal prevalence of the 7-day period will also be calculated. Generalized estimating equations will be used for the investigation at the cluster level. A log-binomial model will be used for calculating the incidence rate of diarrhea. The Random Effects model will be used, taking account of between-cluster variation based upon the assumption that there are cluster-level effects. Per-protocol analysis also will be conducted only as a contingency measure in cases where the coverage of latrines remains at an unexpectedly low level by the time when the intervention is completed. However, if we employ a per-protocol analysis, we cannot quantify the effects of well-equipped latrines, as this would break the comparability between the control and intervention groups obtained from randomization. Instead, we would note that per-protocol analysis is only an auxiliary analysis method to give additional background information. In addition, multi-level analysis will be conducted to investigate whether children living in the households without latrines could benefit from herd protection when a *quartier* reaches a certain level of latrine coverage (for instance, 70%).

## Discussion

This trial aims to investigate the effects of well-equipped latrines when reaching up to a universal or sufficient level, rather than exploring the behavioral factors for latrine improvement. Recent studies [14, 15] with the same purpose failed to reach a universal or sufficiently high coverage level despite employing rigorous methodology; therefore the results can hardly indicate the true effects of high latrine coverage. For this reason, to come up with a solution to achieve the sufficiently high level of coverage, we paid close attention to the lessons learned from a study conducted in Bangladesh [31], whose results suggested that subsidies could increase latrine ownership both in subsidized and unsubsidized households. In the trial, material subsidies will be provided to all households in the intervention arm who complete the latrine improvement activities (e.g. digging a 1.5 m-deep pit, installing a superstructure and roof) in contrast to the selective subsidy provision in previous studies [14, 15]. It is critically important to mobilize community members to reach sufficient sanitation coverage. SNHR officials take on the responsibility to educate community members on the importance of improved latrines and encourage them to participate in the collective efforts to increase latrine coverage at the *quartier* level, and mobilize locally available materials and unskilled labor voluntarily to construct their household latrine.

**Fig. 6:**
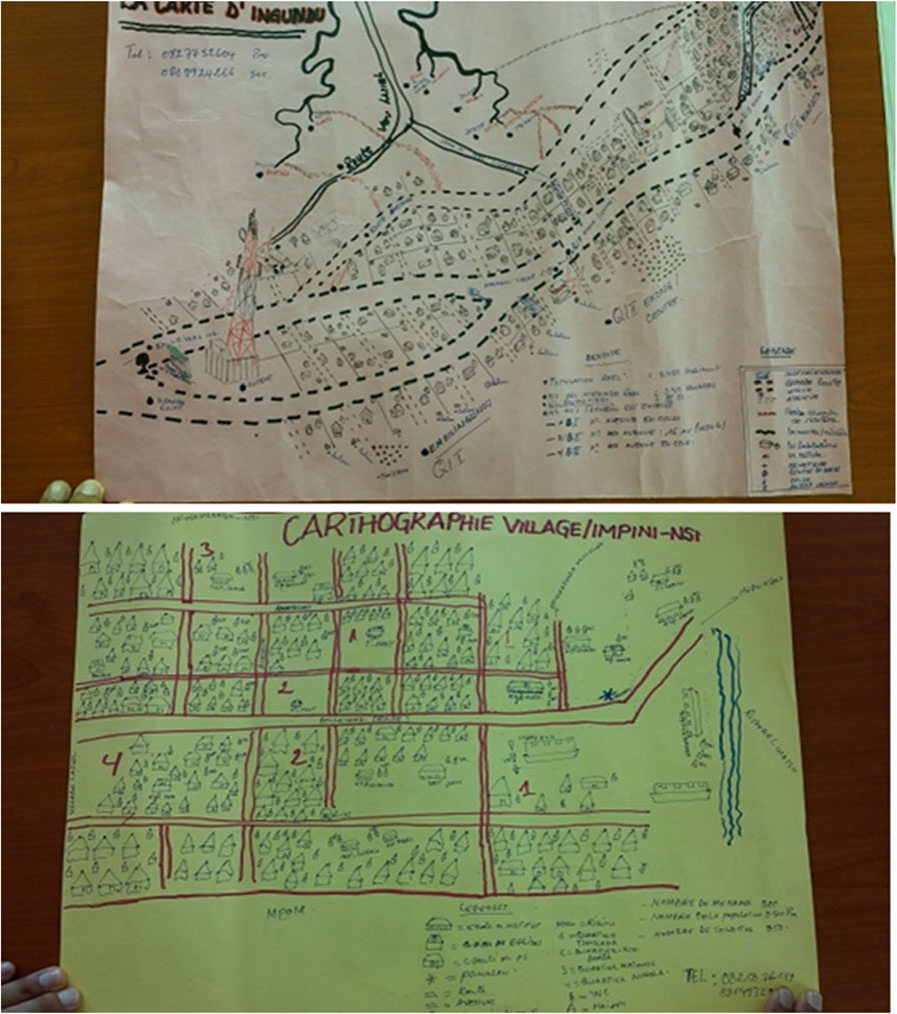
Sanitation map of Ingundu quartier (Draft version, June 2015), Sanitation map of ImpiniNsi quartier (Draft version, June 2015) - are correctly shown

The project will be effectively monitored through the ‘Sanitation Map’. The WASH committee will visit each household to monitor the latrine uptake progress and to draw the Sanitation Map for each *quartier* on a monthly basis (Fig. 6). The WASH committee drew a draft version of a Sanitation Map on a pilot basis in the run-up to the intervention (June 2015), and it was found that the WASH committee understood the objectives of sanitation mapping well enough for the project implementer to monitor progress. The Sanitation Map will be continuously updated through the early, middle and final stage of project implementation, both in the intervention and control arms.

All the 7286 households on the Sanitation Map will be assigned an identity number and marked with a color. They will be marked red when the household latrine does not improve, green when the latrine improves, and yellow when the latrine is under construction. The Sanitation Map can help the WASH committee as well as community members check the progress not only of the *quartier* they belong to, but also of the other groups. Community members will be educated on the herd protection effects of well-equipped latrines and also the importance of the 80% target for latrine coverage. If a *quartier* reaches 80% well-equipped latrine coverage, it will be certified as a ‘Healthy *quartier*’ and rewarded by the project implementer. The sanitation mapping is expected to create peer pressure within and among the *quartiers,* sparking ‘healthy’ competition that will expand the rate of coverage.

In addition, the Sanitation Calendar is to be used as an effective method of tracking diarrhea for a registered child. With the Sanitation Calendar, the incidence density of diarrhea can be calculated by dividing diarrhea cases by observed child numbers multiplied by the specific duration observed (e.g. week, month, etc.). This would be a more effective tool since it would cause less fatigue among the respondents, who would otherwise be asked to provide information about their children’s diarrheal episodes on a daily basis by interviewers. Assuming that the incidence of diarrhea among children is three times a year on average [32], the number of days marked ‘O’ in mothers’ diaries will not be very high. As for the duration of diarrhea, it was estimated at 10–20 days on average. Except for the days marked with ‘O,’ all the other days will be considered ‘X’. The Sanitation Calendar allows the day-to-day observation of diarrhea and resolves the limitations associated with the small number of clusters in the trial. Previous studies were unable to identify the effect of improved sanitation on diarrheal duration because they relied on the period prevalence of reported diarrhea. The utilization of the Calendar will enable us to explore a relationship between sanitation improvements and diarrhea and investigate the extent to which well-equipped and appropriately managed latrines affect disease duration. The measurement relying on caregivers’ reports, which are subjective in nature, is one of the limitations of this study. As we will not be able to conduct laboratory tests to assess children’s diarrhea incidence, there may be under-reporting or over-reporting of diarrhea. Despite this risk, we do not expect any significant difference of reporting patterns between the people of the intervention and control *quartiers*.

This trial is an efficacy trial because the focus is on proving the theory that universal sanitation coverage will reduce diarrhea, and thus, the interventions are designed to deliver high/universal latrine coverage with more certainty than an effectiveness trial implemented at scale [14, 15]. We intentionally developed a small-scale trial to ensure the successful results of sanitation promotion. There is a clear benefit to a small-scale project because the intensive execution of sanitation promotion is likely to be more effective in reaching the universal sanitation coverage in the setting of a small-scale project. Indeed, it has been reported that more sanitation promotion activities yielded success in small scale projects than in large scale projects [33]. However, the sufficient level of coverage is not always guaranteed in any circumstances, which represents another limitation of this study.

Sanitation coverage is defined as the proportion of households who have access to a well-equipped latrine that meets the six criteria specified by the UNICEF Healthy Village Programme in the DRC. In estimating the coverage, however, the access to communal latrines or using a neighbor’s latrine is excluded to focus on latrine usage at the household level. To avoid information bias or courtesy bias among the members of the WASH committee, the progress of latrine improvement will be observed and photographed with digital cameras provided to the WASH committee (June, 2015). Based on the photos taken by the committee, the supervisors will verify the improvement status. Two-meter-long sticks will be provided to the WASH committee to measure the depth of a pit. Latrine utilization will be assessed by the latrine condition, observing various elements such as odor, wet feces in the pit, a spider-web at the entrance, or a worn path to the latrine.

The degree of latrine improvement will be scored according to the well-equipped latrine criteria. This will allow us to assess whether and how the diminishing effect of well-equipped latrines on diarrhea incidence among children under four differs depending on the degree of latrine improvement. Previous studies [14, 15] exploring the health benefits of sanitation have not paid attention to the possibility that the effect of latrines might vary depending on the degree of their improvement. Although a recent trial [21] reported an increase in access to one’s own latrine from 33 to 64.8% in rural Mali, they found no effect of a community-led sanitation improvement on the children’s diarrhea. Based on the 5-f theory, the Joint Monitoring Programme defines an “improved sanitation facility as one that hygienically separates human waste from human contact,” highlighting the importance of accounting for improvement status of latrines. In the case of pit latrines, the existence of a slab and hand-washing facilities, pit-hole depth and number of flies could be considered criteria for identifying the improved status of latrines. Applying these criteria, the proportion of improved latrines in Pickering et al. (i.e., latrines with concrete slabs) would be reduced to less than 30%. Focusing solely on the presence or access to any latrine type may mask the genuine health benefits of improved latrines.

The study hypothesis was not disclosed to people subject to the trial, though the general objectives were explained to them when obtaining informed consent. Thus, people are highly unlikely to be aware of the arm to which they belong because the sanitation intervention will continue to be executed in the control arm during the second phase. We thus surmise that the underreporting of diarrheal prevalence among the caregivers with a household latrine will not be a serious problem in this study. However, the limitation of not blinding still remains in this study since having a latrine is explicit in itself and many of the WASH committee members in the control arms were aware of the intervention even from the beginning due to their participation in the community lottery activity for random allocation.

Since the trial’s randomization process yielded remarkably balanced results between the two arms, and since no other intervention is expected to roll out until February 2017, any differences observed in diarrheal incidence can be attributable to latrine improvement.

For this trial, keeping the Sanitation Calendar is crucial to documenting the progress toward the primary endpoint. A supervisory visit to the WASH committee will be made at least twice every month to encourage the mother or caregiver to record diarrheal incidence on a daily basis, in an unbiased manner. On this visit, the WASH committee will photograph the Sanitation Calendar to confirm whether it is being kept well, which will be verified by supervisors’ (WSA and SNHR sanitation experts) direct observations during the randomly sampled household visit. During the early stage of the intervention, in July and August 2015, strong emphasis will be placed on the importance of appropriately recording outcomes in the Sanitation Calendar. Accordingly the supervisory visit to randomly sampled households will be strengthened to help people get accustomed to recording in the Calendar in a desirable manner. The WASH committee will convene a community meeting to review the Sanitation Calendar on a regular basis and significant errors, if any, will be corrected.

Considering the alternative sanitation policies evaluation result [31], the non-discriminative distribution of material subsidies contingent upon voluntary preparation of some materials for latrine improvement and a commitment of labor may increase the uptake of well-equipped latrines within a short time period, with a strong social multiplier by increasing the ownership. This trial is expected to provide valuable information on the resource allocation of sanitation improvement by determining the effects of improved sanitation on diarrheal reduction for children. The findings of this study will be relevant to populations where improved water and sanitation coverage is minimal.

## Additional file

Additional file 1: Multilingual abstracts in the five official working languages of the United Nations. (PDF 765 kb)

## Abbreviations

KECO: Korea Environment Corporation
KOICA: Korea International Cooperation Agency
MDGs: Millennium Development Goals
SNHR: National Service for Rural Water Supply in the Democratic Republic of the Congo
UNICEF: United Nations International Children’s Fund
WASH committee: Water, sanitation and hygiene committee
WSA: Water and Sanitation for Africa
