# Supplementary material for: Effects of improved sanitation on diarrheal reduction for children under five in Idiofa, DR Congo: a cluster randomized trial
Source: Infect Dis Poverty. 2017 Sep 19;6:137. doi: 10.1186/s40249-017-0351-x (PMC5604412; doi:10.1186/s40249-017-0351-x)
Supplement: Additional file 1: — Multilingual abstracts in the five official working languages of the United Nations. (PDF 765 kb) [file 40249_2017_351_MOESM1_ESM.pdf]

Translation of the abstract into the five official working languages of the United Nations

تأثيرات تحسين الصرف الصحي على الحد من الإسهال للأطفال دون سن الخامسة في ايدويفا، جمهورية الكونغو الديمقراطية: تجربة مجموعة عشوائية

تشأ سينغمان، لي جون، سيو دونجسيك، بارك بيونغ مان، بول مانسيانجي، برنارد كابوري، جي جيروم نكاي مولاكوب-ياجو، أونوريه مينكا فاماسولو

ملخص

الخلفية: الافتقار إلى المياه المأمونة ومرافق الصرف الصحي يسهم في تفشي الإسهال في كثير من البلدان النامية.

الأساليب: <bx/>هذه الدراسة تصف تصميم تجربة مجموعة عشوائية في ايديوفا، "جمهورية الكونغو الديمقراطية وتسعى إلى الحصول على أدلة عن تأثير مرافق الصرف الصحي المحسنة على الإسهال لدى لأطفال دون سن الرابعة. 276 حي، 18 حي <ex/> <bx/> خصصت عشوائياً للتدخل أو لذراع التحكم. أخذت عينات ل 720 أسرة وتم تسجيل أصغر طفل دون سن الرابعة من كل أسرة لهذه الدراسة. نقطة النهاية الأساسية لهذه الدراسة هو الإصابة بالإسهال، التفشي والمدة الزمنية عند الأطفال دون سن الخامسة.

مناقشة: <bx/>ستقدم إعانات مادية فقط للأسر التي تقوم بحفر الحفر بالإضافة إلى بناء البنية الفوقية والسقف، بغض النظر عن مستوى دخلهم. هذه الدراسة تستخدم "مفكرة صحية" حيث بإمكان الأم في كل أسرة معيشية القيام يومياً بتسجيل نوبات الاسهال لدى طفلها دون سن الرابعة . المذكرات تتيح دراسة تأثير التدخل في مجال المرافق الصحية في فترة الإسهال ويحل أيضا محدودية العدد القليل من المجموعات في التجربة.

بالإضافة إلى ذلك، سيتم رصد المشروع من خلال '"مخطط الصرف الصحي"'، الذي تسجله جميع الأسر المعيشية في منطقة الدراسة، بما في ذلك أذرع التحكم والتدخل. لتجنب تحيز المعلومات أو تحيز المجاملة، سوف تؤخذ صوراً للمراحيض خلال زيارة سكان البيت لها ، وسيحدد مشرف امتصاص المراحيض المجهزة تجهيزاً جيداً استناداً إلى الصور. هذا يقلل من احتمال التحيز الاستذكاري او النقص او الافراط في تقدير الاسهال، وهو من أوجه القصور الرئيسية في الدراسات السابقة.

تسجيل التجربة: أقرتالدراسة "من قبل مجلس المراجعة المؤسسية" لكلية الصحة العامة، جامعة كينشاسا (ESP/CE/040/15؛ 13 نيسان/أبريل عام 2015)، وسجلت "كمعيار دولي لتجربة مدارة بشكل عشوائي" (أعطى: 10419317) في 13 مارس 2015.

Translated from English version into Arabic by Maha Husaini, through

TRANSLATORS
WITHOUT BORDERS

刚果民主共和国伊迪奥法地区改善卫生条件对五岁以下儿童腹泻减少的影响：一项集群随机试验

Seungman Cha, JaeEun Lee, DongSik Seo, Byoung Mann Park, Paul Mansiangi, Kabore Bernard, Guy Jerome Nkay Mulakub-Yazho, Honore Minka Famasulu

摘要

引言：由于缺乏安全饮水和卫生设施使得腹泻在许多发展中国家非常猖獗。

方法：本研究描述了一项在刚果民主共和国伊迪奥法地区开展的集群随机试验，并寻求改善卫生条件对四岁以下儿童腹泻影响的证据。 在 276 个研究点中，18 个点随机分配为干预组或控制

组。 对 720 户家庭进行抽样，并对每户中最小的 4 岁以下儿童进行注册并加入本研究。 研究的主要终点是五岁以下儿童的腹泻发病率，患病率和持续时间。
讨论：本研究仅向完成厕所挖坑并加盖上层建筑和屋顶的家庭提供物质补贴，不论其收入水平如何。本研究采用卫生日历以便每个家庭的母亲每天可以记录其四岁以下孩子的腹泻发作情况。日记可以帮助检查卫生干预对腹泻持续时间的影响，也可以解决试验中少数集群的局限性。
此外，本项目将通过“卫生地图”对研究区的所有已登记的住户进行监测，包括控制组和干预组。为了避免信息偏倚或礼貌偏差，家访期间将拍摄厕所照片，主管根据照片确定厕所的好坏。这样可以减少召回偏倚和对腹泻过度或不足估计的可能性，而这些是以往研究的主要限制。
试验注册：本研究由金沙萨大学公共卫生学院机构审查委员会批准（ESP / CE / 040/15; 2015 年 4 月 13 日），并于 2015 年 3 月 13 日注册为国际标准随机对照试验（ISRCTN：10419317）。

Translated from English version into Chinese by Xin-Yu Feng, edited by Pin Yang

CHINESE CENTER FOR DISEASE CONTROL AND PREVENTION
NATIONAL INSTITUTE OF PARASITIC DISEASES

Résultats de l'amélioration de l'assainissement sur la réduction des maladies diarrhéiques chez les enfants de moins de cinq ans à Idiofa, en République Démocratique du Congo: un essai randomisé par groupe

Seungman Cha, JaeEun Lee, DongSik Seo, Byoung Mann Park, Paul Mansiangi, Kabore Bernard, Guy Jerome Nkay Mulakub-Yazho, Honore Minka Famasulu

Résumé

Contexte: Le manque d’eau potable et l'absence de moyens d'assainissement contribuent à la recrudescence des maladies diarrhéiques dans de nombreux pays en développement.
Approches: Cette étude décrit les caractéristiques de la conception d’un essai randomisé par groupes à Idiofa, en République démocratique du Congo, réunissant les preuves de l'impact de l’amélioration des moyens d'assainissement sur les maladies diarrhéiques chez les enfants de moins de quatre ans. Des 276 quartiers, 18 quartiers ont été répartis au hasard en groupe témoin ou en groupe expérimental. 720 ménages ont été choisis et le plus jeune des enfants âgé de moins de quatre ans dans chacun de ces ménages a été inscrit pour cette étude. Les principaux critères de l’étude sont le nombre de cas des maladies diarrhéiques, la prévalence et la durée chez les enfants de moins de cinq ans.
Discussion: Seuls les ménages qui auront fait construire une fosse ajoutée d'une superstructure et d'un couvercle, bénéficieront de subventions significatives, quelque soit leur niveau de revenu. Cette étude utilise un calendrier d'hygiène pour que la mère de chaque ménage puisse enregistrer chaque jour les périodes de diarrhée chez son enfant âgé de moins de cinq ans Le calendrier permet d’examiner l'impact des interventions en matière d’assainissement sur la durée de la diarrhée et résout également la limitation du petit nombre de groupes pendant l'essai.
De plus, le projet se fera par le biais de la « carte de l’assainissement », sur laquelle sont inscrits tous les ménages dans la zone d’étude, y compris le groupe de contrôle et le groupe expérimental. Afin d’éviter tout parti pris ou "toute courtoisie de circonstance", les photographies des latrines seront effectuées lors des visites dans les familles et un agent de supervision déterminera le bon

fonctionnement d'absorption des déchets des latrines à l'aide des photographies. Cela réduit la possibilité d'erreurs de mémoire et de sous-estimation ou de sur-estimation des cas de diarrhée, qui étaient les principales réserves des études précédentes.
Enregistrement des essais: L'étude a été approuvée par la Commission institutionnelle de l'Ecole de santé publique, université de Kinshasa (ESP/CE/040/15 ; 13 avril 2015) et enregistré sous un numéro standard international pour les essais contrôlés randomisés (ISRCTN : 10419317) le 13 mars 2015.

Translated from English version into French by veromarie, through

TRANSLATORS
WITHOUT BORDERS

Влияние санитарных условий на сокращение случаев диареи у детей в возрасте до пяти лет в Идиофе, Демократическая Республика Конго: Рандомизированное кластерное исследование

Сеунгман Ча (Seungman Cha), ЖеУн Ли (JaeEun Lee), ДонгСик Сео (DongSik Seo), Биоунг Манн Парк (Byoung Mann Park), Пол Мансианги (Paul Mansiangi), Каборе Бернард (Kabore Bernard), Гай Джером Нкай Мулакуб-Яжо (Guy Jerome Nkay Mulakub-Yazho), Оноре Минка Фамасулу (Honore Minka Famasulu)

Краткое изложение содержания

Исходные данные: Нехватка безопасной воды и санитарных условий способствует распространению диареи во многих развивающихся странах.
Методы: Данное исследование описывает проект рандомизированного кластерного исследования в Идиофе, Демократическая Республика Конго, в поиске доказательств влияния улучшенных санитарных условий на диарею у детей в возрасте до четырех лет. Из 276 *кварталов*, 18 *кварталов* были распределены по случайной схеме между экспериментальными или контрольными группами. Было выбрано 720 семейств, и самые маленькие дети, в возрасте до четырех лет, из каждой семьи были зарегистрированы для этого исследования. Основной целью исследования является коэффициент заболеваемости, уровень распространения и продолжительность диареи у детей в возрасте до пяти лет.
Изучение вопроса: Материальные субсидии будут предоставлены только тем семействам, которые завершат строительство колодца, надстройки и крыши, независимо от уровня их доходов. Данное исследование предполагает использование Санитарного Календаря, чтобы мать каждого семейства могла ежедневно записывать случаи диареи ее ребенка, возрастом меньше четырех лет. Дневник даёт возможность исследовать влияние санитарного вмешательства на продолжительность диареи, а также решает проблему недостаточного количества кластеров в ходе исследования.
Кроме того, проект будет контролироваться с помощью «Санитарной Карты», на которой будут зарегистрированы все семейства в зоне исследования, включая также экспериментальные и

контрольные группы. Во избежание ошибок или необъективности, во время посещений семейств будут сделаны фотографии уборных, и, основываясь на фотографии, инспектор определит уровень оборудования уборной. Это уменьшит возможность предвзятости и недо - или пере - оценки диареи, что было основной проблемой в предыдущих исследованиях.
Регистрация исследования: Исследование было одобрено Институциональным наблюдательным комитетом Школы общественного здравоохранения, Университет Киншасы, (ESP/CE/040/15; 13 апреля 2015) и зарегистрировано в качестве Международного стандартного рандомизированного контролируемого исследования (ISRCTN: 10419317) 13 марта 2015 года.

Translated from English version into Russian by Viktorija Putneva, through

TRANSLATORS
WITHOUT BORDERS

Efectos de la mejora de la red de saneamiento sobre la reducción de la diarrea en niños menores de cinco años en Idiofa, República Democrática del Congo: Ensayo aleatorio grupal

Seungman Cha, Jae Lee, DongSik Seo, Parque Byoung Mann, Paul Mansiangi, Kabore Bernard, Guy Jerome Nkay Mulakub-Yazho, Honore Minka Famasulu

Resumen

Antecedentes: La falta de agua potable y de red de saneamiento contribuye a que la diarrea se extienda en muchos países en vías de desarrollo.
Métodos: este estudio describe el diseño de un ensayo aleatorizado por conglomerados en Idiofa, República Democrática del Congo, que buscan la evidencia del impacto de la mejora de la red de saneamiento sobre la diarrea de niños menores de cuatro años. De los 276 , 18*barrios* fueron asignados al azar a la intervención o control armado. se tomó una muestra de 720 hogares y se registraron un menor de cuatro años de cada hogar para este estudio. El objetivo primario del estudio es la incidencia, predominio y duración de la diarrea en los niños menores de cinco años.
Discusión: Se ofrecerán subsidios materiales sólo a aquellos hogares que terminen la excavación de un pozo más una superestructura y la construcción de tejado, independientemente de su nivel de ingresos. Este estudio emplea un calendario de saneamiento para que la madre de cada hogar puede grabar los episodios diarreicos de sus hijos menores de cuatro años en una base diaria. El diario permite el examen del efecto de la intervención sanitaria sobre la duración de la diarrea y también soluciona el límite de la pequeña cantidad de agrupaciones en el ensayo.
Además, el proyecto será supervisado a través del mapa de saneamiento, en el que estarán registrados todos los hogares en el área de estudio, incluyendo el control y la intervención de armas. Para evitar sesgos de información o sesgo de cortesía, se tomarán fotos de la letrina durante la visita familiar, y un supervisor determinará el buen equipamiento de la misma basada en las fotos. Eso reduce la posibilidad de recoger el sesgo y la sobreestimación o infraestimación de la diarrea, que era la limitación principal de estudios previos.

Registro del ensayo: El estudio fue aprobado por el Consejo De Revisión Institucional de la Escuela de Salud Pública, Universidad de Kinshasa ((ESP/CE/040/15; 13 de Abril de 2015) y registrado como Ensayo de Grado Internacional Controlado Aleatoriamente (ISRCTN: 10419317) el 13 de Marzo de 2015.

Translated from English version into Spanish by ISABEL SANLLEHI, through

TRANSLATORS WITHOUT BORDERS
